# Seasonal to Inter-Annual Variability of Primary Production in Chesapeake Bay: Prospects to Reverse Eutrophication and Change Trophic Classification

**DOI:** 10.1038/s41598-020-58702-3

**Published:** 2020-02-06

**Authors:** Lawrence W. Harding, Michael E. Mallonee, Elgin S. Perry, W. David Miller, Jason E. Adolf, Charles L. Gallegos, Hans W. Paerl

**Affiliations:** 1 Department of Atmospheric and Oceanic Sciences, University of California, Los Angeles, Los Angeles, California 90095 United States; 2 Interstate Commission on the Potomac River Basin, United States Environmental Protection Agency, Chesapeake Bay Program Office, 410 Severn Avenue, Annapolis, Maryland 21403 United States; 3 Statistics Consultant, 377 Resolutions Rd., Colonial Beach, Virginia 22443 United States; 4 U.S. Naval Research Laboratory, 4555 Overlook Ave., SW, Washington, D.C. 20375 United States; 5 Department of Biology, Monmouth University, West Long Branch, NJ 07764 United States; 6 Smithsonian Institution, Smithsonian Environmental Research Center, 647 Contees Wharf Road, Edgewater, Maryland 21037 United States; 7 Institute of Marine Sciences, University of North Carolina at Chapel Hill, 3431 Arendell Street, Morehead City, North Carolina 28557 United States

**Keywords:** Ecology, Environmental sciences, Ocean sciences

## Abstract

Estuarine-coastal ecosystems are rich areas of the global ocean with elevated rates of organic matter production supporting major fisheries. Net and gross primary production (NPP, GPP) are essential properties of these ecosystems, characterized by high spatial, seasonal, and inter-annual variability associated with climatic effects on hydrology. Over 20 years ago, Nixon defined the trophic classification of marine ecosystems based on annual phytoplankton primary production (APPP), with categories ranging from “oligotrophic” to “hypertrophic”. Source data consisting of shipboard measurements of NPP and GPP from 1982 to 2004 for Chesapeake Bay in the mid-Atlantic region of the United States supported estimates of APPP from 300 to 500 g C m^−2^ yr^−1^, corresponding to “eutrophic” to “hypertrophic” categories. Here, we developed generalized additive models (GAM) to interpolate the limited spatio-temporal resolution of source data. Principal goals were: (1) to develop predictive models of NPP and GPP calibrated to source data (1982 to 2004); (2) to apply the models to historical (1960s, 1970s) and monitoring (1985 to 2015) data with adjustments for nutrient loadings and climatic effects; (3) to estimate APPP from model predictions of NPP; (4) to test effects of simulated reductions of phytoplankton biomass or nutrient loadings on trophic classification based on APPP. Simulated 40% decreases of euphotic-layer *chl-a* or TN and NO_2_ + NO_3_ loadings led to decreasing APPP sufficient to change trophic classification from “eutrophic’ to “mesotrophic” for oligohaline (OH) and polyhaline (PH) salinity zones, and from “hypertrophic” to “eutrophic” for the mesohaline (MH) salinity zone of the bay. These findings show that improved water quality is attainable with sustained reversal of nutrient over-enrichment sufficient to decrease phytoplankton biomass and APPP.

## Introduction

**Figure 1:**
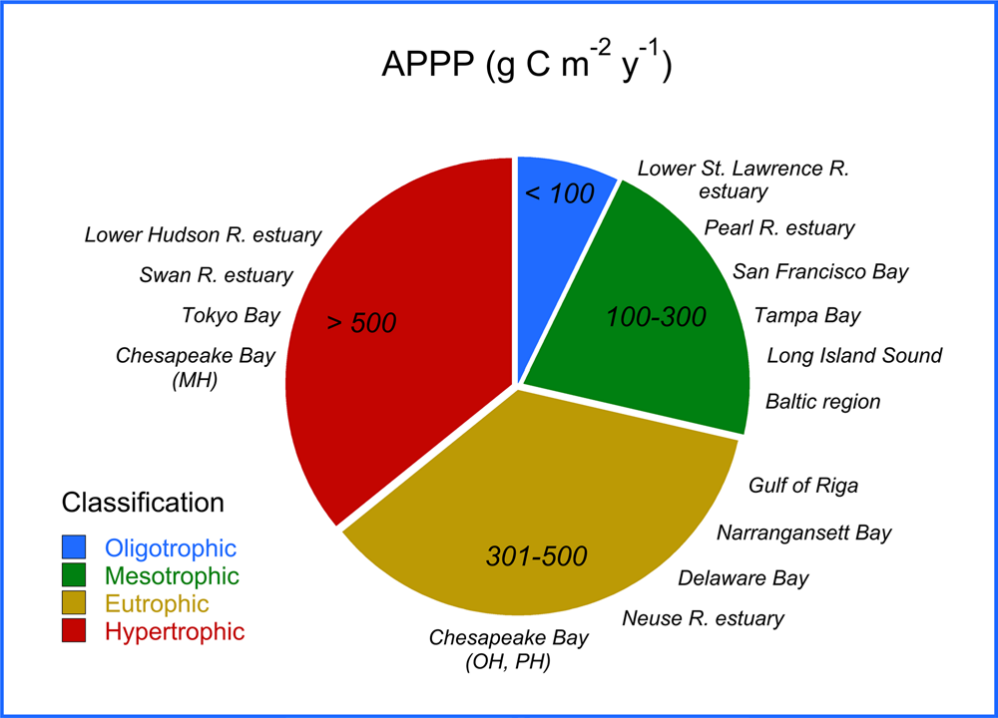
Trophic classification presented by Nixon (1995) based on APPP (g C m^−2^ y^−1^) including examples for selected estuarine-coastal ecosystems^4^.

Annual phytoplankton primary production (APPP) accounts for ~50 petagrams (=50 × 10^12^ kg) of net primary production (NPP) in the oceans each year, half the global total for oceanic and terrestrial ecosystems according to a comprehensive review by Chavez *et al*.^1^. Among ocean provinces, estuarine-coastal ecosystems have been characterized as biogeochemical “hot spots” by Cloern *et al*.^2^ based on high contributions to APPP. In 1995, Nixon^3^ classified marine ecosystems as “oligotrophic” to “hypertrophic” based on APPP, with several estuaries in the mid-Atlantic region of the United States ranking toward the high end of the range (Fig. 1). Important advances in understanding phytoplankton dynamics in estuarine-coastal ecosystems followed a Chapman Conference of the American Geophysical Union (AGU) convened in Rovinj, Croatia in 2004, culminating in comprehensive global syntheses that highlighted long-term trends of biomass, floral (=taxonomic) composition, and APPP^2,4–6^.

**Figure 2:**
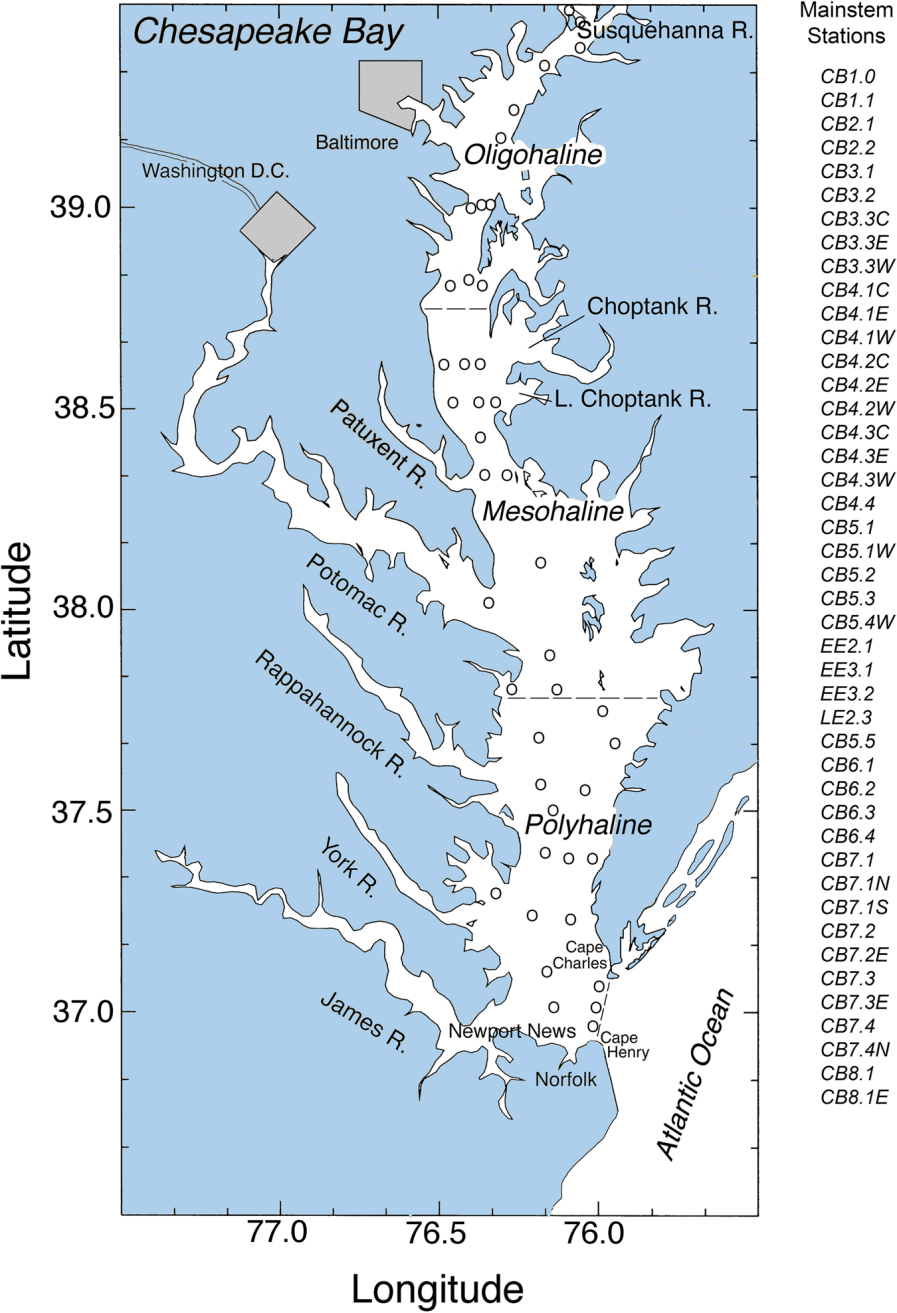
Map of study site showing major rivers, cities, salinity zones, and water-quality sampling stations in Chesapeake Bay.

Nearly 20 years ago, we published depth-integrated models of net and gross primary production (NPP, GPP) for Chesapeake Bay (Fig. 2)^7^. These models were based on an approach by Behrenfeld and Falkowski^8^, the Vertically Generalized Production Model (VGPM), calibrated with long-term measurements of ^14^C-assimilation in the bay from 1982 to 2004^7^. Spatial, seasonal, and inter-annual variability of NPP and GPP in the bay was not well-defined prior to our studies, limiting the ability to resolve inter-annual variability of APPP^9–15^. We recently quantified climatic effects on water-quality properties including *chl-a*, Secchi depth, and oxidized nitrogen (nitrite plus nitrate = NO_2_ + NO_3_) using statistical models with terms for freshwater flow, salinity, and nutrient loadings to distinguish variability from long-term trends^16–18^. Here, we apply a similar approach to NPP and GPP, leading to improved spatio-temporal resolution of APPP sufficient to resolve inter-annual variability.

Recognition of eutrophication as a pressing issue in Chesapeake Bay stimulated individual studies dating to the 1970s^9–26^, and long-term monitoring of water-quality properties initiated in the mid-1980s (Chesapeake Bay Program, US Environmental Protection Agency, http://www.chesapeakebay.net). Combined results define an annual cycle of phytoplankton biomass dominated by a spring bloom of centric diatoms following the winter-spring freshet of the Susquehanna River, with integrated, water-column chlorophyll*-a* (*chl-a*) reaching ~1000 mg m^−2^ from April to mid-May. North-south gradients of light and nutrients driven by freshwater discharge deliver buoyancy, nutrients, and suspended particulate matter to the bay, strongly affecting the timing, position, and magnitude of the spring bloom, as reviewed by Malone^14^.

Seasonal warming leads to sea-surface temperatures (SST) ranging from 18 to 20 °C by late spring to early summer as persistent density stratification sets up. Deposition of organic material originating from the spring-bloom provides the substrate to fuel microbial metabolism, eventually leading to depletion of dissolved oxygen (DO) beneath the pycnocline, and regeneration of nutrients to support maximum NPP in summer. A transition in the phytoplankton community occurs from May to June following the sinking of spring-bloom diatoms, with a decrease of integrated, water-column *chl-a* to <200 mg m^−2^ ^14^. The summer flora is primarily composed of smaller, <20 µm diatoms, flagellated cells, including dinoflagellates, chrysophytes, cryptophytes, and non-motile picoplankton, such as cyanobacteria and other small, <3 µm cells, supporting the annual maximum of NPP from July to August^27,28^.

Nutrient over-enrichment of the bay led to a 5- to 10-fold increase of surface *chl-a* for the polyhaline (PH) salinity zone, and a 1.5- to 2-fold increase for oligohaline and mesohaline (OH, MH) salinity zones from the 1950s to the 1990s^20,21^. These increases were stimulated by eutrophication after World War II, evident in upward “trajectories” of total nitrogen (TN) and NO_2_ + NO_3_ loadings^29,30^. Seasonal and inter-annual variability of freshwater discharge underlies spatio-temporal variability of water-quality properties, superimposed on historical changes and complicating the detection of long-term trends. We used statistical models to distinguish the eutrophication signal from variability associated with climatic effects, documenting the doubling of flow-adjusted TN and NO_2_ + NO_3_ loadings from 1945 to the early 1980s^16–18^. Newer studies followed this approach to adjust for climatic effects on water quality, phytoplankton biomass, floral composition, and NPP^16–19^.

Despite numerous studies of plankton ecology in Chesapeake Bay, limited spatio-temporal resolution of NPP and GPP restricts our ability to define inter-annual variability of APPP. To address this limitation, we developed statistical models of NPP and GPP based on earlier depth-integrated models^7,31^, including an expanded set of predictor variables to account for nutrient loading and climatic effects on hydrology^16–18^. Principal goals were: (1) to develop predictive models of NPP and GPP calibrated to source data (1982 to 2004); (2) to apply the models to historical (1960s, 1970s) and monitoring (1985 to 2015) data with adjustments for nutrient loadings and climatic effects; (3) to estimate APPP from model predictions of NPP; (4) to test effects of simulated 40% reductions of phytoplankton biomass or nutrient loadings on trophic classification based on APPP. Targeting these goals allowed us to test the hypothesis that nutrient reductions adopted by Chesapeake Bay jurisdictions would lead to reduced APPP and a change of trophic classification.

## Methods

### Cruises

Data were collected on 78 cruises on four research vessels from March 1982 to November 2004. All stations we occupied to measure NPP and GPP are depicted in Fig. 1. Data from a subset of these cruises were analyzed previously^7^. Experimental protocols for measuring NPP and GPP were specific to individual projects identified by acronyms in Table 1. CB cruises (1982–83) consisted of an initial transect for horizontal mapping to determine salinity, temperature, *chl-a*, nutrient, and turbidity gradients and to select stations for measuring NPP. ProPhot and FITS cruises (1987–88) and LMER PROTEUS cruises (1989–94) occupied stations along a north-south transect to map water-quality properties and to measure NPP and ancillary properties. NASA cruises (1993–94) occupied three stations per cruise in the lower bay, plume, and adjacent shelf waters interspersed with mapping legs to measure NPP, GPP, and ancillary properties. LMER TIES cruises (1995–2000) sampled stations at 0.5° latitudinal increments, and additional stations lateral to the north-south axis, for a total of nine to 27 stations per cruise. LMER TIES cruises also included legs for horizontal and vertical mapping, *in-situ* sampling of phytoplankton, zooplankton, and fish, towed-body sampling with an instrumented SCANFISH (Geological & Marine Instrumentation), and continuous underway measurements of water-quality properties using the ship’s Serial ASCII Instrumentation Loop (SAIL) system. EPA, NASA, and NSF cruises (2001–2004) used the same protocols as TIES cruises.

**Table 1:** Summary of cruises, research vessels, and dates for measurements of ^14^C-assimilation in simulated *in-situ* incubations for samples collected at 716 stations on 78 cruises from 1982 to 2004.

Cruise	Research Vessel	Date	Cruise	Research Vessel Date	
CB-1	*Cape Hatteras*	Mar-82	NASA 94-02	*Cape Henlopen*	Apr-94
CB-2	*Cape Hatteras*	Jun-82	LMER 94-02	*Cape Henlopen*	Apr-94
CB-3	*Cape Hatteras*	Nov-82	LMER 94-05	*Cape Henlopen*	Jul-94
CB-4	*Cape Hatteras*	Mar-83	NASA 94-05	*Aquarius*	Jul-94
ProPhot-23	*Ridgley Warfield*	Apr-87	LMER 94-08	*Cape Henlopen*	Oct-94
ProPhot-24	*Ridgley Warfield*	May-87	TIES 98-01	*Cape Henlopen*	Apr-98
FITS-13	*Ridgley Warfield*	Jul-87	TIES 98-02 bp	*Cape Henlopen*	Jul-98
FITS-15	*Ridgley Warfield*	Aug-87	TIES 98-02	*Cape Henlopen*	Aug-98
ProPhot-25	*Ridgley Warfield*	Mar-88	TIES 98-03	*Cape Henlopen*	0ct-98
ProPhot-26	*Ridgley Warfield*	Apr-88	TIES 99-01	*Cape Henlopen*	Apr-99
ProPhot-27	*Ridgley Warfield*	May-88	TIES 99-02	*Cape Henlopen*	Jun-99
LMER 89-1	*Cape Henlopen*	Feb-89	TIES 99-02raz	*Cape Henlopen*	Jul-99
LMER 89-2	*Cape Henlopen*	Mar-89	TIES 99-02 bp	*Cape Henlopen*	Jul-99
LMER 89-3	*Cape Henlopen*	Apr-89	TIES 99-03	*Cape Henlopen*	Oct-99
LMER 89-4	*Cape Henlopen*	May-89	TIES 00-01	*Cape Henlopen*	Apr-00
LMER 89-5	*Cape Henlopen*	Jun-89	TIES 00-02	*Cape Henlopen*	Jul-00
LMER 89-6	*Cape Henlopen*	Jul-89	TIES 00-03	*Cape Henlopen*	Oct-00
LMER 89-7	*Cape Henlopen*	Aug-89	BIO 01-01	*Cape Henlopen*	Apr-01
LMER 89-8	*Cape Henlopen*	Sep-89	BIO 01-02	*Cape Henlopen*	Aug-01
LMER 89-9	*Cape Henlopen*	Nov-89	BIO 01-03	*Cape Henlopen*	Oct-01
LMER 90-3	*Cape Henlopen*	Apr-90	BIO 02-01	*Cape Henlopen*	Apr-02
LMER 90-4	*Cape Henlopen*	Apr-90	BIO 02-02	*Cape Henlopen*	Jul-02
LMER 90-5	*Cape Henlopen*	May-90	BIO 02-03	*Cape Henlopen*	Oct-02
LMER 90-6	*Cape Henlopen*	May-90	BIO 03-01	*Cape Henlopen*	Apr-03
LMER 90-7	*Cape Henlopcn*	Jul-90	MOVE 08-03	*Cape Henlopen*	Aug-03
LMER 90-8	*Cape Henlopen*	Aug-90	BIO 03-02	*Cape Henlopen*	Oct-03
LMER 90-11	*Cape Henlopen*	Nov-90	SGER 11-03	*Cape Henlopen*	Nov-03
LMER 91-3	*Cape Henlopen*	Apr-91	ACE 04-01	*Cape Henlopen*	Apr-04
LMER 91-4	*Cape Henlopen*	May-91	BIO 04-01	*Cape Henlopen*	Apr-04
LMER 91-6	*Cape Henlopen*	Jul-91	ACE 04-02	*Cape Henlopen*	Apr-04
LMER 91-7	*Cape Henlopen*	Aug-91	ACE 04-02B	*Cape Henlopen*	May-04
LMER 91-9	*Cape Henlopen*	Sep-91	ACE 04-03B	*Cape Henlopen*	May-04
LMER 91-10	*Cape Henlopen*	Oct-91	ACE 04-04	*Cape Henlopen*	Jun-04
LMER 91-11	*Cape Henlopen*	Oct-91	ACE 04-04B	*Cape Henlopen*	Jun-04
LMER 91-11	*Cape Henlopen*	Oct-91	ACE 04-04 C	*Cape Henlopen*	Jul-04
LMER 92-03	*Cape Henlopen*	Apr-92	BIO 04-02	*Cape Henlopen*	Jul-04
LMER 92-06	*Cape Henlopen*	Jul-92	ACE 04-05	*Cape Henlopen*	Aug-04
LMER 92-09	*Cape Henlopen*	Oct-92	ACE 04-06	*Cape Henlopen*	Aug-04
LMER 93-06	*Cape Henlopen*	Oct-93	BIO 04-03	*Cape Henlopen*	Sep-04

### Horizontal transects

Continuous underway sampling on horizontal transects was used to characterize distributions of water-quality properties. Some methods used on horizontal transects were described previously^7^. Instrumentation was specific to the vessel used. For CB, ProPhot, and FITS cruises on the *Cape Hatteras* and *Ridgely Warfield* (1982–1988), discrete samples were spaced 4–5 km along transects. Yellow Springs Instruments model 33 salinometer and model 57 oxygen meter were used for mapping on CB cruises on the *Cape Hatteras* (1982–1983). Interocean, Inc. Inductive Conductivity and Temperature Indicator (ICTI) was used on ProPhot and FITS cruises on the *Ridgely Warfield* (1987–1988). The NASA cruise on the *Aquarius* (1994) used a SeaBird conductivity-temperature-depth-fluorescence-oxygen instrument package (CTDFO_2_) in pump-through mode. NSF LMER PROTEUS, TIES, and NASA, Biocomplexity, and SGER cruises on the *Cape Henlopen* (1989–2004) used the ship’s flow-through SAIL system for surface mapping, with SeaBird sensors for salinity and temperature, and Turner Designs model 10 fluorometers for *chl-a* and turbidity. Instrument readings were calibrated for all transects using periodic grab samples.

### Vertical profiles

Vertical profiles of salinity, temperature, dissolved oxygen (DO), and *chl-a* fluorescence were determined from hydro-casts at a set of stations spaced 10–20 km along horizontal transects. Some of the methods to obtain vertical profiles of these properties were described previously^7^. Hydrocasts were made with Yellow Springs salinity and DO meters (see above) on 1982–1983 cruises, a Sea Bird model 9 CTDFO_2_ on 1987–1988 cruises, and a Neil Brown Mark III CTDFO_2_ on 1989–2004 cruises. Discrete samples were collected in Niskin bottles to calibrate fluorometers and DO meters. Surface sunlight during the day (E_0_ = downwelling irradiance, photosynthetically available radiation = PAR) was measured with a Li-Cor quantum meter model 190 S (or equivalent) coupled to a Li-Cor model 550 or 1000 integrator. The sensor was mounted near the deck incubators we used to measure simulated *in-situ* NPP and GPP. Diffuse light attenuation coefficient for PAR (K_PAR_) was determined from vertical profiles with a submersible Li-Cor quantum meter model 188B with a 192S sensor or equivalent for CB, ProPhot, FITS, and LMER PROTEUS, TIES, and NASA cruises. Secchi depths (m) were determined for all stations and cruises. Euphotic-layer depth (Z_p_) was estimated as the depth to which 1% of E_0_ penetrated based on vertical profiles of downwelling irradiance (E_z_), or from Secchi depth using calibration regressions^32^. NSF LMER TIES and NASA cruises also conducted vertical profiles with a Biospherical Instruments multi-spectral environmental radiometer (MER-2040/41) to measure K_PAR_ and spectral light attenuation.

### Water-quality properties

*Chl-a* was determined using spectrophotometric and fluorometric measurements on acetone extracts (90%) of particulate material collected by vacuum filtration onto glass-fiber filters (Whatman GF/F or equivalent, 0.3–0.8 µm nominal pore sizes). Spectrophotometric *chl-a* was derived from trichromatic equations applied to absorbances measured on a Beckman DK-2 or equivalent, and fluorometric *chl-a* was measured on a Turner model 110, 111, or Turner Designs model 10 calibrated by spectrophotometry^20,21^. Secchi depth was the depth at which a 30-cm white disk became invisible when lowered over the side of the research vessel. NO_2_ + NO_3_ was measured using analytical methods documented by the EPA/CBP^33,34^ following protocols described by D’Elia *et al*.^35^.

### Simulated *in-situ* incubations

NPP was measured at 723 stations from 1982 to 2004, and GPP at 525 stations from 1995 to 2004. Whole-water samples were collected in Niskin bottles mounted on a rosette sampler at sunrise at a depth of 0.5 to 1.0 m, contents were pooled in a darkened carboy, and dispensed to 125–150 ml glass incubation bottles. The euphotic layer is well mixed in the bay and *chl-a* is homogenously distributed in the upper 5–10 m. NPP and GPP were determined using ^14^C-sodium bicarbonate uptake in deck incubators cooled with flowing surface water (±1 °C of *in-situ* temperatures). 2 to 5 µCuries of ^14^C-sodium bicarbonate (ICN Pharmaceuticals, Inc., or Amersham Searle, Inc.) were added to each incubation bottle. Total ^14^C-sodium bicarbonate activity was determined for a time-zero aliquot from one of the incubation bottles, and from a small amount of stock isotope added to scintillation cocktail made basic with NaOH (Aquasol, New England Nuclear, Inc., or equivalent). Incubation bottles were exposed to a range of sunlight levels using neutral density screens providing 100 (no screens), 58, 34, 21, 11, 4 and 1% transmission to simulate seven depths in the euphotic layer. Dark uptake was measured in an opaque bottle and used as a tare value to adjust uptake in illuminated bottles.

NPP on cruises from 1982 to 1983 was measured in 3–4 h incubations repeated four times during the photoperiod, with additional bottles incubated throughout the night. NPP on cruises from 1987 to 2004 was measured in 24-h incubations, and GPP on cruises from 1995 to 2004 was measured in 4–6 h incubations. Concurrent measurements using ^14^C and O_2_ methodologies confirmed this approach accurately estimated NPP and GPP^7^. Duplicate 25–150 ml subsamples (depending on phytoplankton biomass) were withdrawn at the end of these periods and filtered onto glass fiber filters (Gelman AE or Whatman GF/F) under low vacuum pressure (<150 mm Hg). Filter pads were rinsed with filtered water of equivalent salinity as the sample and acidified with 0.01 N HCl in a fume hood to remove residual inorganic label. Activities were determined on a Packard Instruments Tri-Carb or model 3320 liquid scintillation counter. Duplicate aliquots were withdrawn from incubation bottles to determine *chl-a* using methods described above. Total CO_2_ was measured by gas-stripping, capture, and analysis on a Beckman model 864 infrared analyzer for 1982–83 cruises, Gran titration for 1987–1988 cruises, and by gas chromatography on a Hach Cable Series 100 AGC for 1989–2004 cruises.

### Computations of NPP and GPP

Equations to compute NPP and GPP combined terms for ^14^C-uptake in lighted bottles tared against non-photosynthetic ^14^C-uptake in dark bottles, divided by the total ^14^C activity added, and multiplied by a discrimination factor of 1.05 for ^14^C vs ^12^C and a term for total CO_2_ (mM) converted to weight (mg m^−3^). The resulting volumetric rates (PP, mg C m^−3^ h^−1^) were used to compute NPP and GPP by converting simulated incubation depths as percent surface irradiance (E_0_) to actual depths based on K_PAR_ from vertical profiles of irradiance (E_d_) or Secchi depth. Multiple-segment trapezoidal integration was applied to PP from the surface to euphotic-layer depth to obtain NPP and GPP. NPP was determined from 24-h incubations and GPP from 4–6 h incubations scaled to the photoperiod using E_0_ during incubations as a proportion of total E_0_ for the day. Optimal photosynthesis, P^B^_opt_, was determined as maximum PP in simulated *in-situ* incubations normalized to *chl-a*. Observed values of log_10_ P^B^_opt_ were binned in 1° increments as a function of SST following the approach of Son *et al*.^31^ and analyzed by polynomial regression. Estimates of log_10_ P^B^_opt_ from these regressions were used as model inputs to predict NPP for historical (1960s, 1970s) and monitoring (1985–2015) programs.

### Freshwater discharge, climatic effects

Daily freshwater discharge from the Susquehanna River (SRF) and monthly cumulative discharge (SUM) were obtained from the U.S. Geological Survey (USGS) (http://md.water.usgs.gov) for the Conowingo Dam gaging station near the head of the bay (latitude 39° 39′ 28.4″, longitude 76° 10′ 28.0″). TN and NO_2_ + NO_3_ loadings were obtained as monthly values (10^6^ kg) from the USGS. We focused on the Susquehanna River as this large river dominates distributions of nutrients and phytoplankton in the main stem bay. Nutrient loadings from other tributaries are significantly reduced by processes within their confines, attenuating effects of lateral inputs on the bay proper^16–18^. Climatic effects were quantified by applying GAM to input files for NPP, GPP, and water-quality properties, including terms for freshwater flow (log_10_ monthly SRF, log_10_ monthly SUM) and salinity. Model predictions of NPP and GPP for low-flow, “dry” conditions were based on flow terms set at 10^th^ percentiles joined by salinity terms at 90^th^ percentiles; mean-flow model predictions were based on flow and salinity terms held constant at their mean values; model predictions for high-flow, “wet” conditions were based on flow terms set at 90^th^ percentiles joined by salinity terms at 10^th^ percentiles.

### Analytical steps

**Figure 3:**
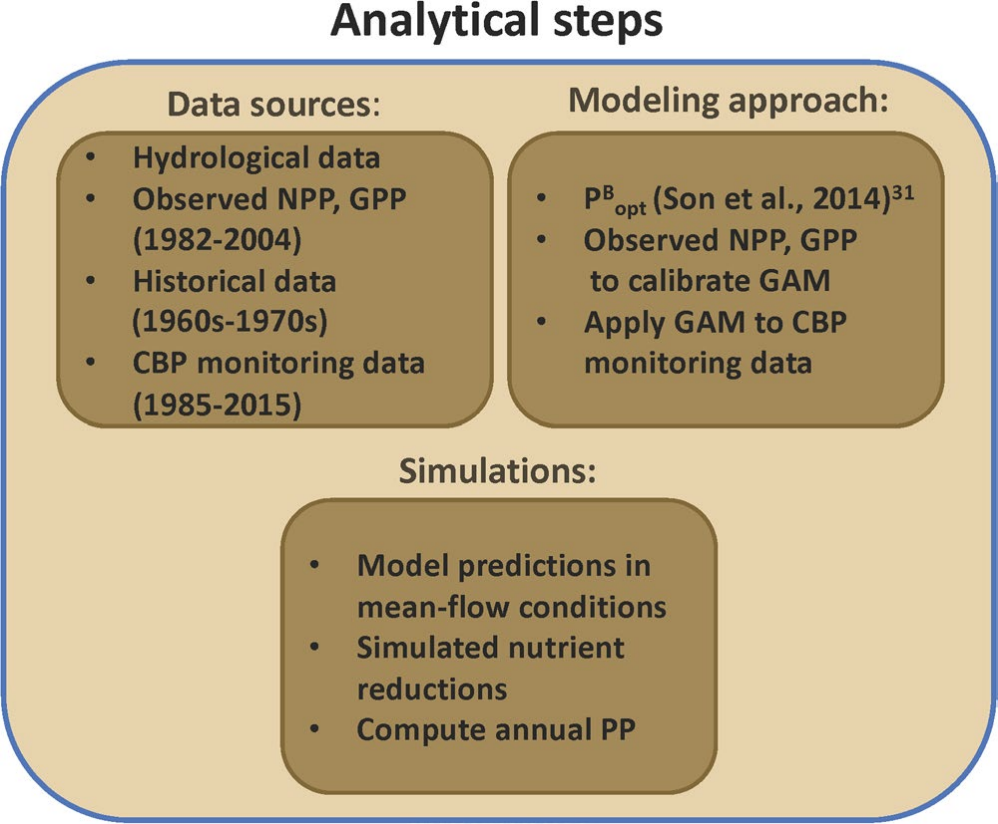
Summary of analytical steps including data sources, modeling approach, and simulations.

A flow-chart of analytical steps, including data sources, modeling approach, and simulations is presented in Fig. 3. Statistical analyses were conducted using “R” v. 3.6.0. Non-linear fits of time-series data were developed using GAM from the “R” package ‘mgcv’, containing functions similar to those designed by T. Hastie in S-Plus, and based on a penalized regression-spline approach including automatic smoothness selection^36–38^. Model predictions of NPP and GPP were adjusted for climatic effects using GAM, as described previously for water-quality properties^16–19^. A comparable approach by Beck and Murphy^39^ compared GAM to weighted regressions of time, discharge and season (WRTDS) developed by Hirsch *et al*.^40^, noting the flexibility of GAM to add relevant predictor variables as a strength of GAM over alternatives.

**Figure 4:**
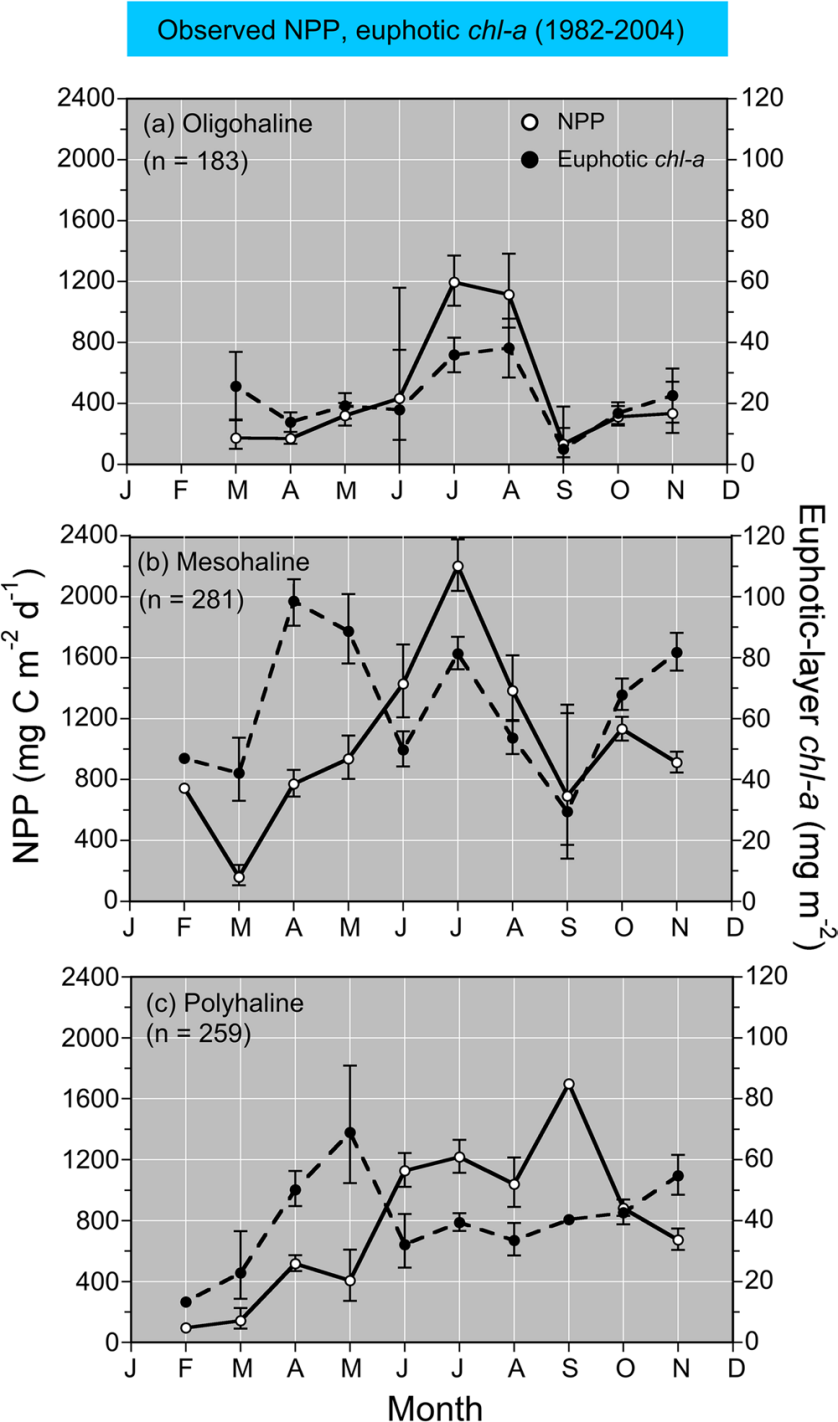
(**a–c**) Mean, monthly (±SE) observed NPP (g C m^−2^ d^−1^) (left ordinate) and euphotic-layer *chl-a* (mg m^−2^) (right ordinate) for oligohaline (OH), mesohaline (MH), and polyhaline (PH) salinity zones.

**Figure 5:**
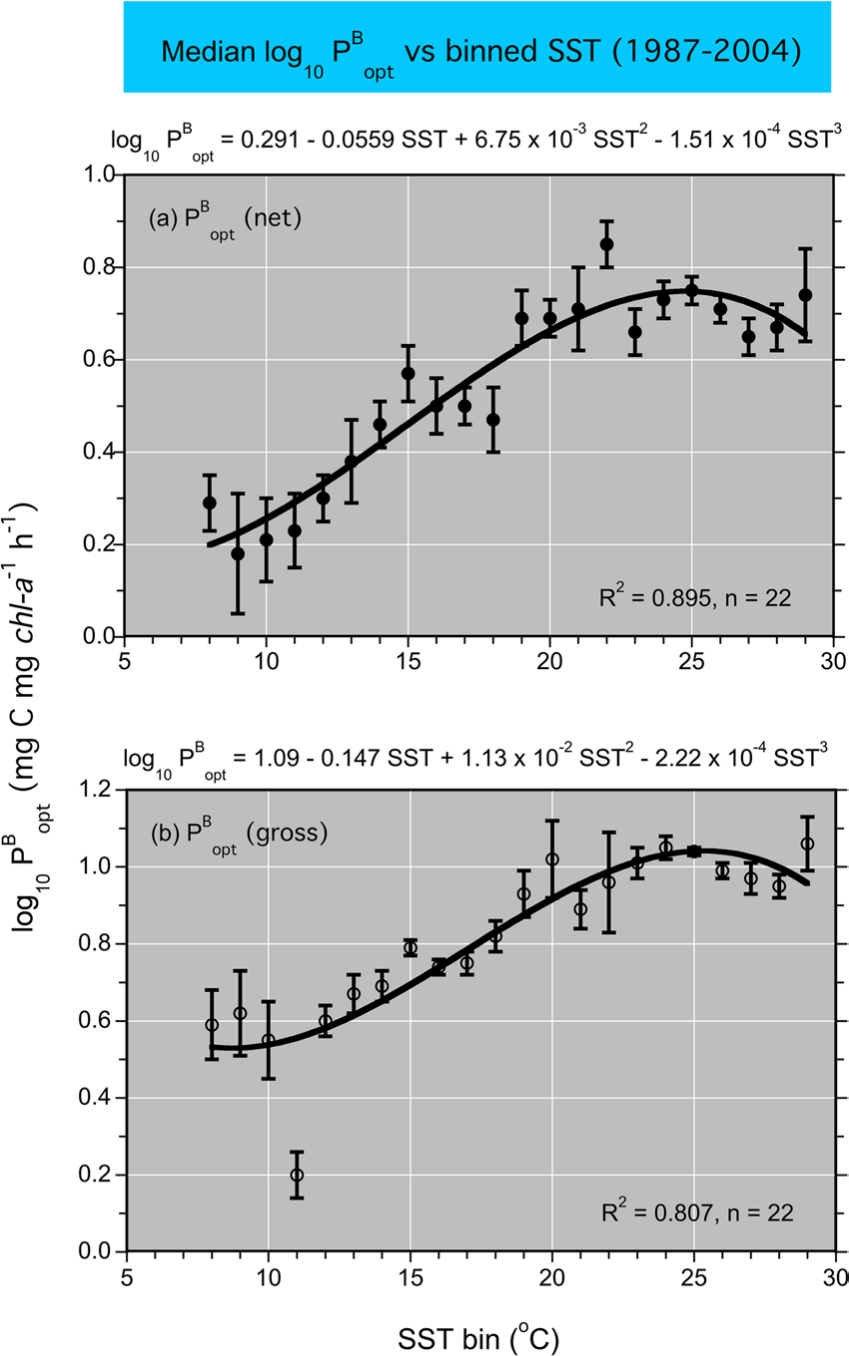
(**a**,**b**) Relationships of log_10_ P^B^_opt_ (net) and (gross) to binned sea-surface temperature (SST). Time-series observations included additional measurements from 2002 to 2004 to update three-order polynomial regressions of Son *et al*.^31^.

**Figure 6:**
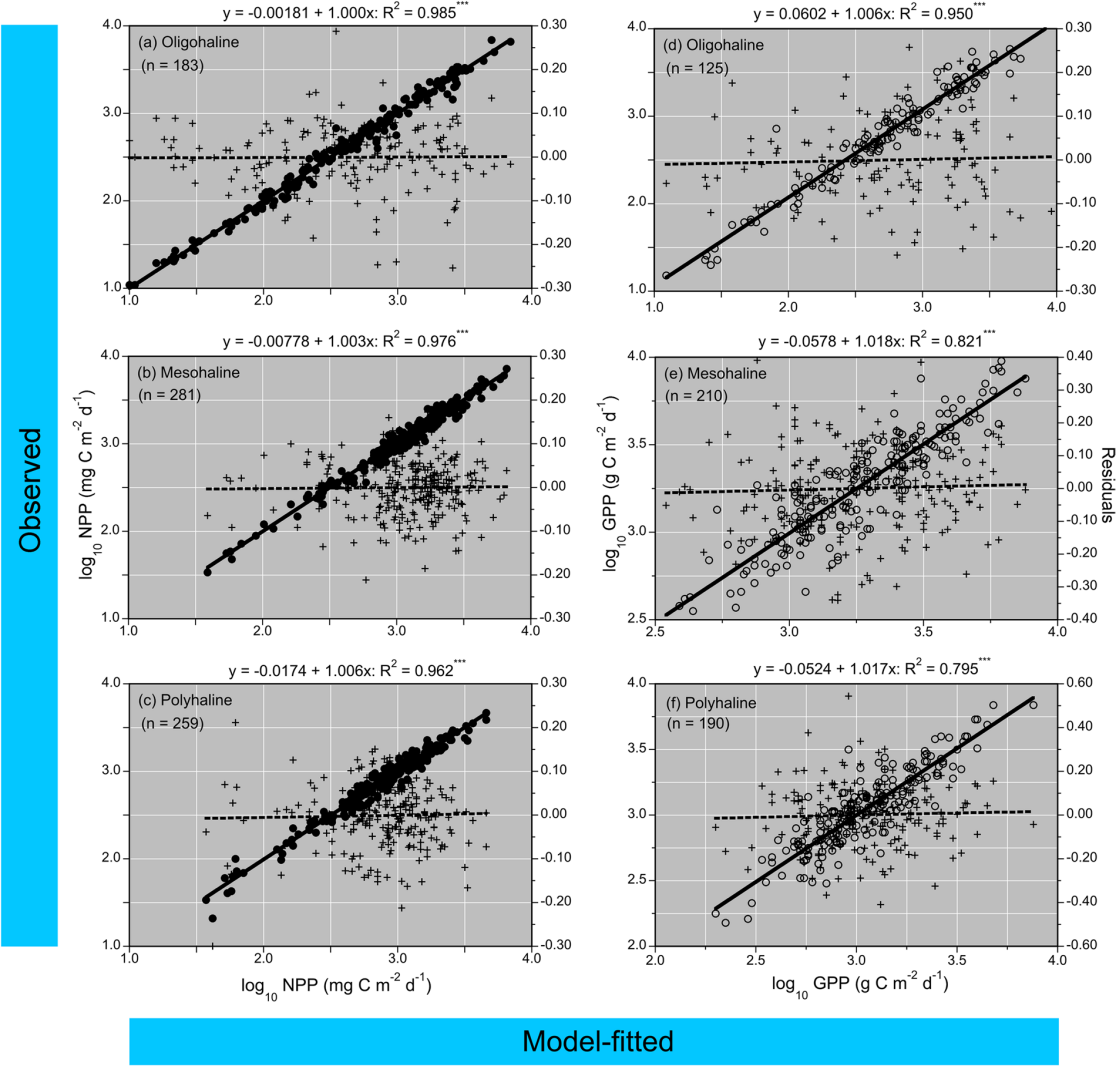
**(a–f)** Simple, linear regressions of observed vs predicted log_10_ NPP and GPP (mg C m^−2^ d^−1^) for OH, MH, and PH salinity zones using generalized additive models (GAM). Source data for GAM were obtained in measurements of simulated *in-situ* ^14^C-bicarbonate assimilation and ancillary properties from 1982 to 2004 (n = 723). Closed circles = log_10_ NPP; open circles = log_10_ GPP; crosses = residuals, dashed lines = simple, linear regressions of residuals vs model-fitted NPP or GPP.

**Figure 7:**
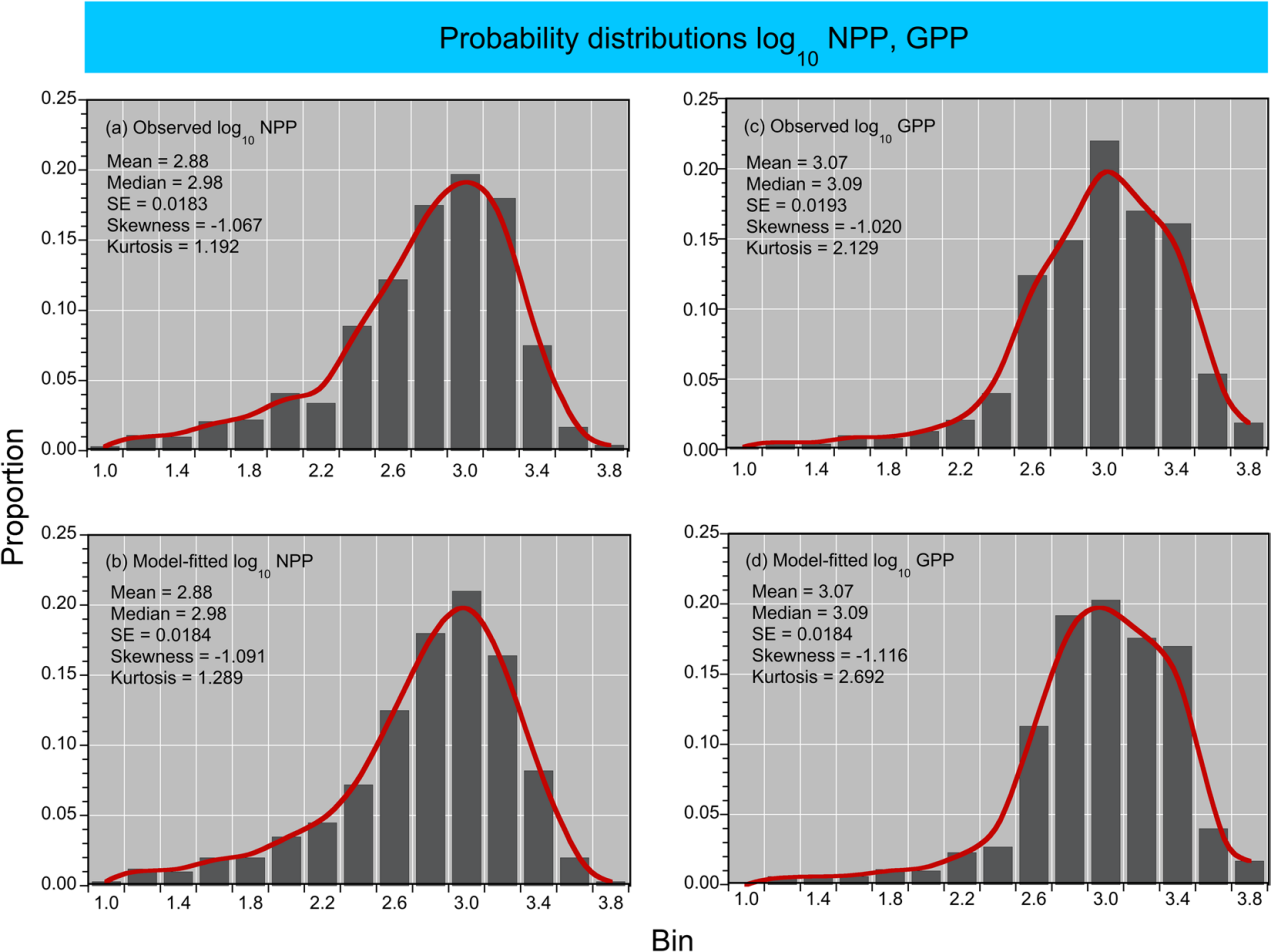
**(a–d)** Probability distributions of observed and predicted daily log_10_ NPP and GPP (g C m^−2^ d^−1^).

**Figure 8:**
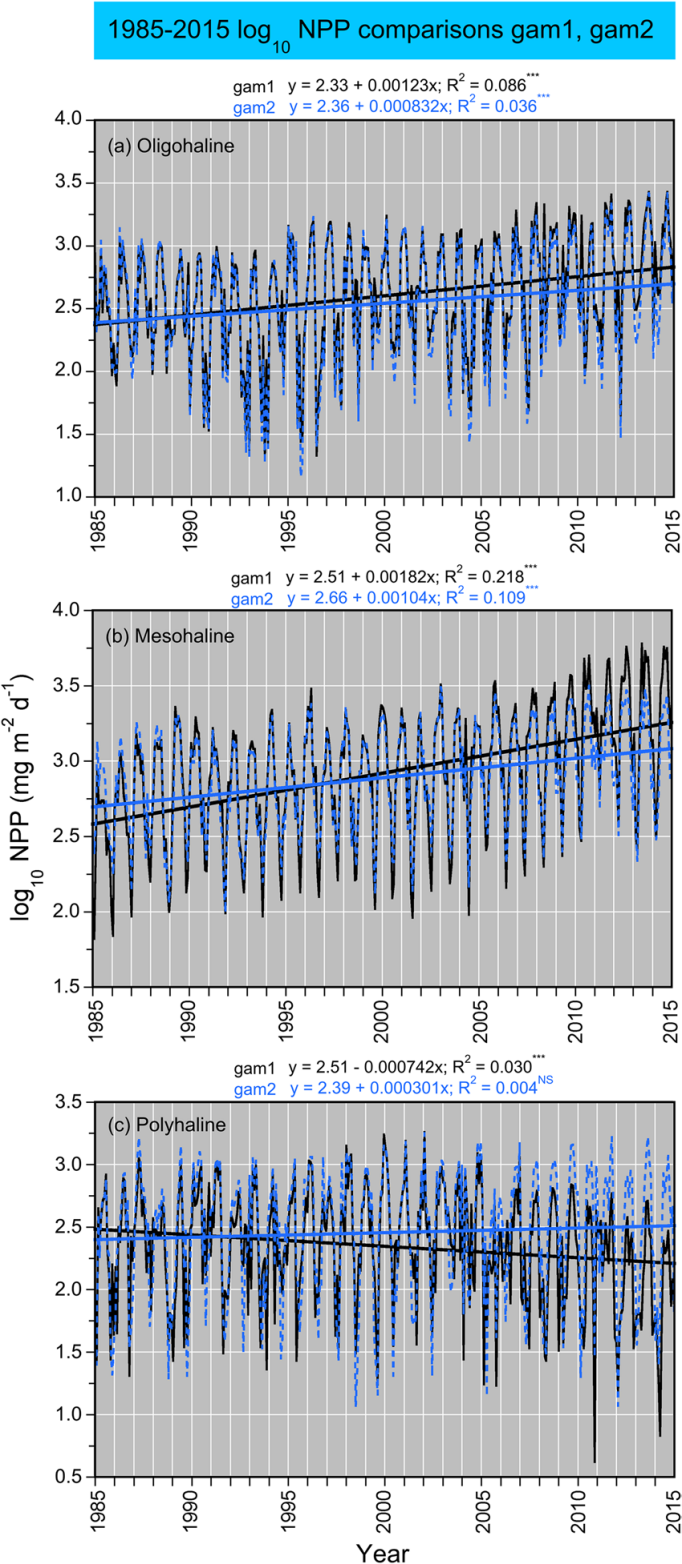
**(a–c)** Comparisons of log_10_ NPP using predictions from generalized additive models (GAM) from 1985 to 2015; gam1 included an predictor variable for the time term “Seq-year” and gam2 omitted this variable (see Table 4).

**Figure 9:**
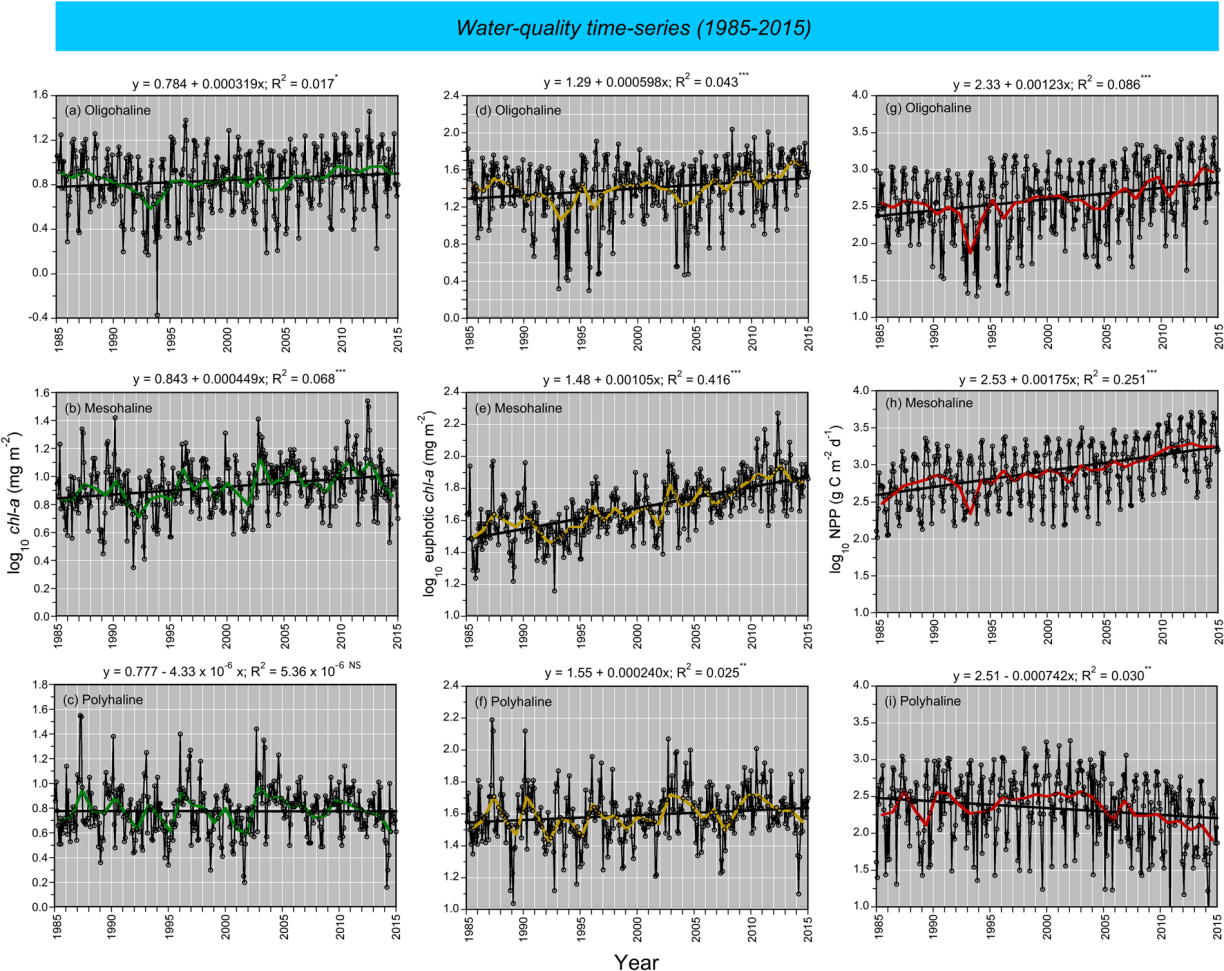
**(a–i)** Mean, monthly surface *chl-a* (mg m^−3^), euphotic-layer *chl-a* (mg m^−2^) and NPP (mg C m^−2^ d^−1^) for OH, MH, and PH salinity zones from 1985 to 2015. Source data for *chl-a* and euphotic-layer *chl-a* were semi-monthly to monthly cruises of the EPA Chesapeake Bay Program (CBP). Model predictions of NPP from gam2 applied to water-quality data from CBP using GAM calibrated with measurements of simulated *in-situ* ^14^C-bicarbonate assimilation (n = 713) and ancillary properties from 1982 to 2004. Climatic effects incorporated using 10^th^, mean, and 90^th^ percentiles of freshwater flow, salinity, and nutrient (TN, NO_2_ + NO_3_) loading as inputs. *Amber dashed lines* = low-flow conditions; *black solid lines* = mean-flow conditions; *blue dashed lines* = high-flow conditions. *Black solid lines* = simple, linear regression of model predictions in mean-flow conditions vs time (months), with regression equations superimposed on each panel.

**Figure 10:**
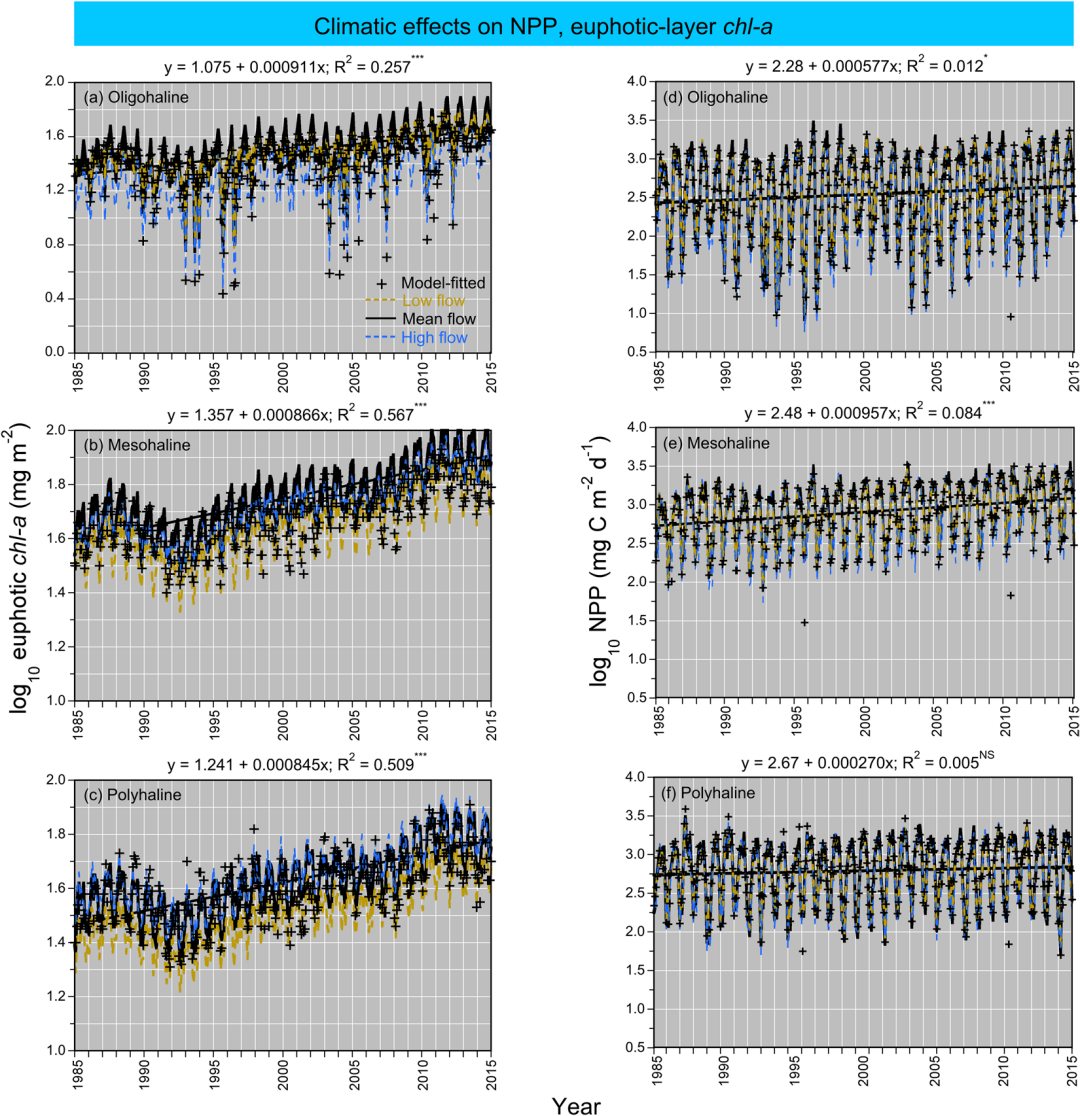
**(a–f)** Model predictions of log_10_ euphotic-layer *chl-a* and log_10_ NPP from 1985 to 2015 using GAM. Flow adjustments were obtained as described in the Fig. 8 caption. *Amber dashed lines* = low-flow conditions; *black solid lines* = mean-flow conditions; *blue dashed lines* = high-flow conditions. *Black solid lines* = simple, linear regressions of model predictions in mean-flow conditions vs time (months), with equations superimposed on each panel.

**Figure 11:**
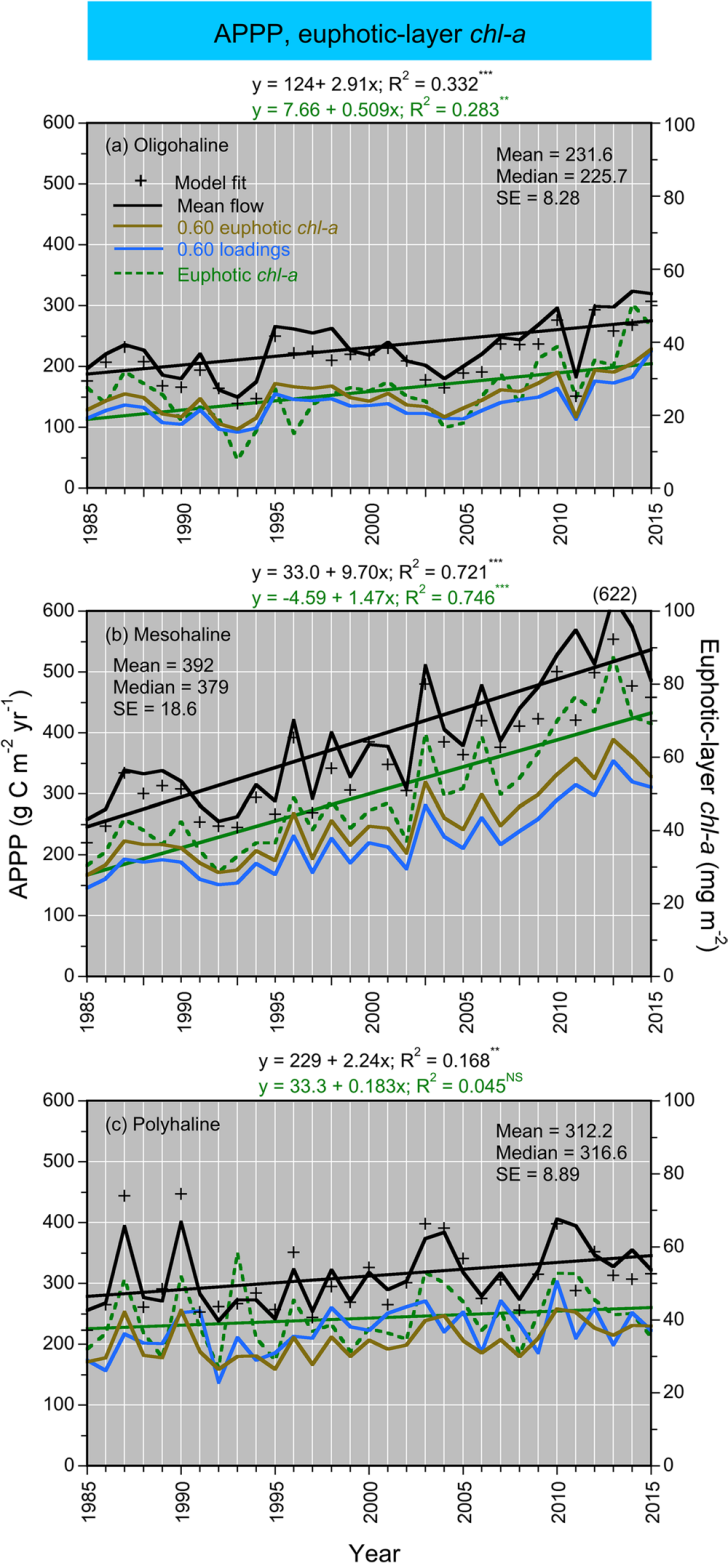
**(a–c)** Time series of APPP and euphotic-layer *chl-a* from 1985 to 2015 for OH, MH, and PH salinity zones. *Crosses* = model predictions in ambient conditions; *black solid lines* = model predictions in mean-flow conditions; *amber solid lines* = model predictions in mean-flow conditions with 40% reduction of euphotic-layer *chl-a*; *blue solid lines* = model predictions with 40% reductions of nutrient (TN, NO_2_ + NO_3_) loadings; *green dashed lines* = mean, annual euphotic-layer *chl-a*.

**Figure 12:**
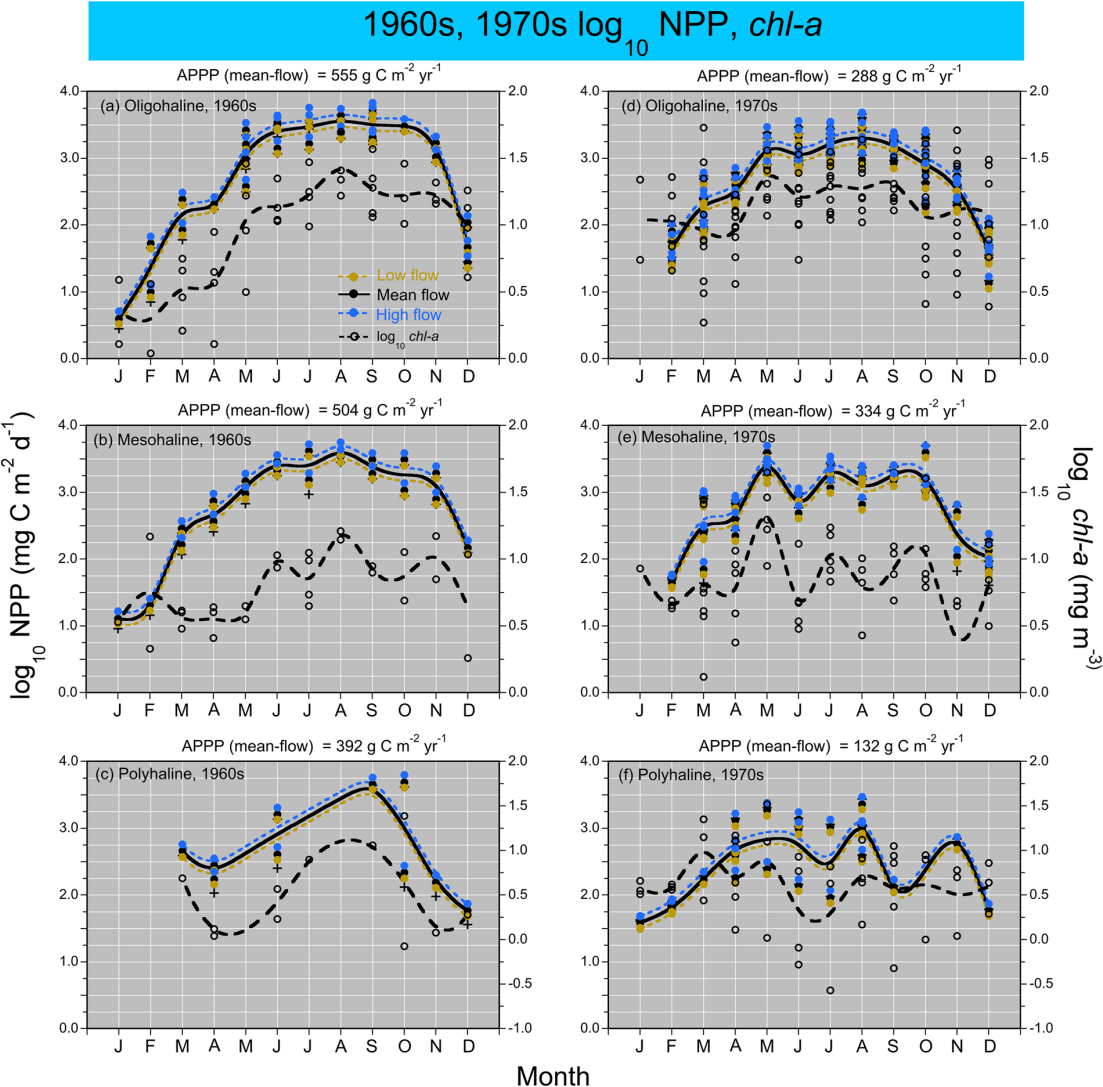
**(a–f)** Mean, monthly log_10_ NPP (left y-axis) and log_10_ *chl-a* (right y-axis) in the 1960s and 1970s for OH, MH, and PH salinity zones. NPP derived using gam5 (Table 4).

Model fits, residuals, flow-adjusted model predictions at monthly increments, adjusted R^2^, generalized cross validation (GCV) score, % deviance explained, p-values for F-statistics, and root mean square error (RMSE) were obtained for each model. Degrees of smoothing (knots = k) were selected by the “R” package ‘mgcv’ to minimize the GCV score, followed by post-hoc adjustments of “k” for individual terms using the function “gam.check”. Graphical presentations were prepared with Kaleidagraph 4.5.2 (Synergy Software, Inc.). These include time series of mean, monthly NPP and euphotic-layer *chl-a* (Fig. 4a–c), polynomial regressions of P^B^_opt_ on SST (Fig. 5a,b), observed vs model-fitted values of log_10_ NPP and log_10_ GPP (Fig. 6a–f), probability distributions of observed and predicted log_10_ NPP and log_10_ GPP (Fig. 7a–d), comparisons of predicted log_10_ NPP from gam1 and gam2 (Fig. 8a–c), time-series of log_10_ *chl-a*, log_10_ euphotic-layer *chl-a*, and model predictions of log_10_ NPP from 1985 to 2015 (Fig. 9a–i), flow-adjusted model predictions of log_10_ euphotic-layer *chl-a* and log_10_ NPP for low-flow, mean-flow, and high-flow conditions (Fig. 10a–f), time series of APPP based on mean-flow model predictions of NPP and simulated reductions of euphotic-layer *chl-a* or TN and NO_2_ + NO_3_ loadings, and observed euphotic-layer *chl-a* (Fig. 11a–c), and historical reconstructions of mean, monthly log_10_ NPP and log_10_ *chl-a* for the 1960s and 1970s with estimates of APPP (Fig. 12a–f).

## Results

### Annual cycles

Spatial and seasonal differences were evident in mean, monthly euphotic-layer *chl-a* and NPP based on shipboard measurements in Chesapeake Bay from 1982 to 2004 (Fig. 4a–c). Seasonal maxima of phytoplankton biomass and production occurred in summer for the OH salinity zone, with euphotic-layer *chl-a* ~40 mg m^−2^ and NPP ~1200 mg C m^−2^ d^−1^ in July and August (Fig. 4a). Contrasting patterns for the MH salinity zone consisted of a well-developed spring bloom with euphotic-layer *chl-a* >80–100 mg m^−2^ in April and May, displaced several months from a summer maximum of NPP >2000 mg m^−2^ d^−1^ in July (Fig. 4b). A second maximum of euphotic-layer *chl-a* from 60–80 mg m^−2^ occurred in fall for the MH salinity zone but was not matched by a maximum of NPP (Fig. 4b). Annual cycles of euphotic-layer *chl-a* and NPP for the PH salinity zone were similar in profile and somewhat lower compared to those for the MH salinity zone, with maxima of euphotic-layer *chl-a* ~60 mg m^−2^ and NPP ~1700 mg m^−2^ d^−1^ in May and September, respectively (Fig. 4c). Table 2 summarizes the statistical properties of shipboard measurements from 1982 to 2004 by salinity zone and season, including Z_p_, salinity, SST, surface *chl-a*, euphotic-layer *chl-a*, NPP, and GPP.

**Table 2:** Statistical properties of shipboard data from 1982 to 2004 used to calibrate models of net and gross primary production (NPP, GPP).

Salinity Zone (units)	Season	Z_p_ (m)	SST (°C)	Salinity	log_10_ *chl-a* (mg m^−3^)	*chla*	L CI, U CI	log_10._ *Euchl-a* (mg m^−2^)	*Euchla*	L CI, U CI	log_10_ NPP (mg C m^−2^ d^−1^)	NPP	L CI, U CI	log_10_ GPP (mg C m^−2^ d^−1^)	GPP	L CI, U CI
OH	Spring	3.45	12.3	3.16	0.764	5.81	(4.57, 7.05)	1.23	17.0	(15.7, 18.3)	2.34	219.9	(218.6, 221.3)	2.51	323.6	(322.2, 325.1)
OH	Summer	4.10	26.4	5.23	0.978	9.50	(8.27, 10.7)	1.56	36.5	(35.2, 37.7)	3.07	1175	(1174, 1176)	3.30	1973	(1971, 1974)
OH	Fall	4.25	16.2	4.65	0.657	4.53	(3.20, 5.87)	1.21	16.0	(14.6, 17.4)	2.47	295.7	(294.3, 297.2)	2.56	363.5	(362.0, 365.0)
MH	Spring	7.15	13.1	11.1	1.13	13.4	(12.2, 14.6)	1.95	88.1	(87.0, 89.3)	2.85	711.7	(710.5, 712.9)	3.07	1180	(1179, 1181)
MH	Summer	6.62	26.7	12.2	1.07	11.8	(10.6, 12.9)	1.85	71.4	(70.3, 72.5)	3.28	1909	(1908, 1910)	3.51	3267	(3266, 3268)
MH	Fall	8.11	18.3	15.0	0.952	8.96	(7.82, 10.1)	1.83	68.1	(66.9, 69.2)	3.02	1056	(1055, 1057)	3.11	1279	(1278, 1280)
PH	Spring	8.74	12.5	19.2	0.823	6.66	(5.40, 7.92)	1.70	49.7	(48.5, 50.9)	2.66	458.9	(457.7, 460.1)	2.85	701.7	(700.5, 702.9)
PH	Summer	9.64	26.4	21.1	0.719	5.24	(4.00, 6.48)	1.58	37.6	(36.5, 38.8)	3.07	1164	(1163, 1165)	3.25	1767	(1765, 1768)
PH	Fall	7.41	18.2	22.8	0.844	6.97	(5.82, 8.13)	1.67	47.1	(45.9, 48.2)	2.90	798.8	(797.8, 799.9)	3.00	1001	(999.8, 1002)

Z_p_ = euphotic-layer depth, SST = sea-surface temperature; *chl-a* = chlorophyll *a*; *Euchl-a* = euphotic-layer *chl-a*; L CI = lower 95% confidence interval; U CI = upper 95% confidence interval.

### Models of P^B^_opt_, NPP, and GPP

P^B^_opt_ was estimated from third-order polynomial regressions of observed log_10_ P^B^_opt_ on binned SST as described by Son *et al*.^31^. These regressions had p < 0.001 and R^2^ > 0.80 (Fig. 5a,b). Estimates of log_10_ P^B^_opt_ from these regressions were combined with input data on water-quality properties to predict NPP and GPP. Table 3 presents a complete list of predictor variables for models of NPP and GPP. Simple, linear regressions of observed vs model-fitted log_10_ NPP and GPP had p < 0.001 for OH, MH, and PH salinity zones (Table 4; Fig. 6a–f). Probability distributions of observed and model-fitted log_10_ NPP and GPP confirmed that the models generated unbiased estimates as model predictions displayed statistical attributes indistinguishable from observations (Fig. 7a–d).

**Table 3:** Predictor variables for generalized additive models (GAM) of net and gross primary production (NPP, GPP) in Chesapeake Bay.

Predictor variables
log_10_ P^B^_opt_ (net or gross)
log_10_ euphotic-layer *chl-a* (or log_10_ *chl-a*)
Salinity zone (categorical)
Sea-surface temperature (SST)
Salinity
K_PAR_ or Z_p_ (light attenuation coefficient or euphotic-layer depth)
Month (numerical, 1–12)
Season (categorical)
Year (sequential from start of time-series)
log_10_ SRF (mean, monthly Susquehanna R. flow)
log_10_ SUM (cumulative, monthly Susquehanna R. flow)
TN loading (monthly or annual)
NO_2_ + NO_3_ loading (monthly or annual)

**Table 4:** Statistics for generalized additive models (GAM) of net and gross primary production (NPP, GPP) in Chesapeake Bay using predictor variables (Table 3) as detailed for **gam2** (Table 5). GCV = generalized cross-validation score; AIC = Akaike information criterion; RMSE = root mean square error.

Property	Time frame	N	R^2^ (adjusted)	% Deviance explained	GCV	AIC	RMSE
**NPP**							
OH	1982–2004	183	0.985	98.9	0.00736	−373.1	0.0757
MH		281	0.971	97.6	0.00537	−663.5	0.0661
PH		259	0.955	96.5	0.00807	−478.8	0.0825
**GPP**							
OH	1995–2004	125	0.961	97.5	0.0217	−141.8	0.1191
MH		210	0.783	82.0	0.0263	−172.4	0.1472
PH		190	0.740	79.5	0.0318	−124.9	0.1575

### Model predictions

Five GAM formulations were developed to accommodate estimates of log_10_ NPP and log_10_ GPP based on availability of data for predictor variables in different time periods (Table 5). We focused primarily on model predictions of log_10_ NPP as these supported estimates of APPP. Model predictions of log_10_ NPP were compared for gam1 and gam2, i.e., model formulations with and without the predictor variable “sequential year” (Seq_year). By omitting Seq_year in gam2, we avoided an assumption that long-term trends in calibration data (1982 to 2004) were unchanged for water-quality properties outside that range (2005 to 2015) that were used to predict log_10_ NPP. Both gam1 and gam2 captured seasonal to interannual variability of log_10_ NPP, with differences in long-term trends expressed in simple, linear regressions of model predictions on year (Fig. 8a–c). Subsequent analyses predicted log_10_ NPP from 1985 to 2015 using gam2 based on water-quality properties as data inputs. An alternate model to gam2 was developed as gam3 to address data limitations for historical periods, using the term log_10_ *chl-a* for biomass in place of log_10_ euphotic-layer *chl-a*. gam3 was further modified to gam4 by omitting the term K_PAR_ and to gam5 by omitting the term for season. These models were applied to input data from the 1960s and 1970s as log_10_ euphotic-layer *chl-a*, K_PAR_, or season were either absent or too sparse to support model predictions with reasonable sample sizes.

**Table 5:** GAM models of net primary production (NPP) for the oligohaline (OH) salinity zone.

**gam1.pp.net.oh** <- gam(log_PP_net ~ s(log_Pbopt_net) + s(log_Euchl) + s(Kpar) + s(Temp) + s(Salin) + s(Month, bs = “cc”, k = 8) + s(Seq_year) + Seq_year + Season + s(LOG_SRF) + s(LOG_SUM) + s(TN_LDG) + s(NO23_LDG), data = PP_OH)^a,b^
**gam2.pp.net.oh** <- gam(log_PP_net ~ s(log_Pbopt_net) + s(log_Euchl) + s(Kpar) + s(Temp) + s(Salin) + s(Month, bs = “cc”, k = 8) + Season + s(LOG_SRF) + s(LOG_SUM) + s(TN_LDG) + s(NO23_LDG), data = PP_OH)^c^
**gam3.pp.net.oh** <- gam(log_PP_net ~ s(log_Pbopt_net) + s(log_Chl) + s(Kpar) + s(Temp) + s(Salin) + s(Month, bs = “cc”, k = 8) + Season + s(LOG_SRF) + s(LOG_SUM) + s(TN_LDG) + s(NO23_LDG), data = PP_OH)^d^
**gam4.pp.net.oh** <- gam(log_PP_net ~ s(log_Pbopt_net) + s(log_Chl) + s(Temp) + s(Salin) + s(Month, bs = “cc”, k = 8) + Season + s(LOG_SRF) + s(LOG_SUM) + s(TN_LDG) + s(NO23_LDG), data = PP_OH)^e^
**gam5.pp.net.oh** <- gam(log_PP_net ~ s(log_Pbopt_net) + s(log_Chl) + s(Temp) + s(Salin) + s(Month, bs = “cc”, k = 8) + s(LOG_SRF) + s(LOG_SUM) + s(TN_LDG) + s(NO23_LDG), data = PP_OH)^d,e^

^a^gam models of NPP for MH and PH salinity zones had the same structures as these models, using input data PP_MH and PP_PH; models based on the all calibration data (1982 to 2004) used input data PP_ALL and added a categorical term for Salzone (OH, MH, PH);

^b^gam models to predict GPP had the same structure as those for NPP with log_Pbopt_gross substituted for log_Pbopt_net;

^b^gam2 models applied to water-quality data from 1985 to 2015 were used to predict NPP from data files WQ_OH, WQ_MH, and WQ_PH; the explanatory variable Seq_year was omitted from these models to avoid extending trends in calibration data (1982 to 2004) to years outside that time frame;

^c^Substituted log_Euchl with log_Chl in gam models to predict NPP to test models using input data that lacked euphotic-layer *chl-a*;

^d^Annual TN and NO_2_ + NO_3_ loadings were replaced with monthly loadings in gam models to predict NPP for historical data, based on the lack of data on Kpar or monthly loadings for the 1960s and 1970s;

^e^Season was omitted as a categorical variable in gam models to predict NPP due to small sample sizes for historical data.

### Water-quality time series

Observed mean, monthly log_10_ *chl-a* (mg m^−3^) (Fig. 9a–c), log_10_ euphotic-layer *chl-a* (mg m^−2^) (Fig. 9d–f), and model predictions of log_10_ NPP (mg C m^−2^ d^−1^) (Fig. 9g–i) are presented for OH, MH, and PH salinity zones from 1985 to 2015. Corresponding statistical properties for these water-quality data are compiled in Table 6. Simple, linear regressions showed upward trends of log_10_ *chl-a* for OH and MH salinity zones (Fig. 9a,b), but no trend for the PH salinity zone (Fig. 9c); log_10_ euphotic-layer *chl-*a showed upward trends for OH, MH, and PH salinity zones (Fig. 9d–f); log_10_ NPP increased for OH and MH salinity zones (Fig. 9g,h), and decreased for the PH salinity zone (Fig. 9i).

**Table 6:** Statistical properties and predicted net primary production (NPP) for water-quality data from 1985 to 2015. NPP FIT = model-fitted NPP using observed flow and salinity; NPP MNS = flow-adjusted NPP using long-term means of freshwater flow and salinity.

Salinity Zone (units)	Season	Z_p_ (m)	Temp (^o^C)	Salinity	log_10_ *chl-a* (mg m^−3^)	*chl-a*	L CI, U CI	log_10_. *Euchl-a* (mg m^−2^)	*Euchl-a*	L CI, U CI	log_10._ NPP FIT (mg C m^−2^ d^−1^)	NPP FIT	L CI, U CI	log_10_ NPP MNS (mg C m^−2^ d^−1^)	NPP MNS	L CI, U CI
OH	Winter	4.04	3.73	5.60	0.630	4.30	(3.17, 5.43)	1.21	16.1	(14.9, 17.3)	1.84	69.8	(68.6, 71.0)	1.83	67.4	(66.2, 68.6)
OH	Spring	3.42	11.6	4.76	0.910	8.09	(6.99, 9.19)	1.42	26.4	(25.2, 27.5)	2.53	341.2	(340.0, 342.4)	2.56	361.9	(360.7, 363.1)
OH	Summer	3.54	26.7	6.90	1.09	12.3	(11.3, 13.4)	1.63	42.8	(41.8, 43.9)	3.10	1264	(1263, 1265)	3.09	1239	(1238, 1240)
OH	Fall	4.25	17.3	7.78	0.780	5.99	(4.89, 7.09)	1.39	24.4	(23.3, 25.5)	2.59	386.9	(385.7, 388.2)	2.71	510.6	(509.4, 511.8)
MH	Winter	5.87	11.7	13.6	0.869	7.40	(6.31, 8.49)	1.66	45.7	(44.6, 46.8)	2.47	291.6	(290.4, 292.7)	2.46	290.6	(289.4, 291.7)
MH	Spring	5.39	9.40	12.1	0.957	9.06	(7.94, 10.2)	1.71	50.9	(49.8, 52.0)	2.87	746.3	(745.2, 747.5)	2.91	812.2	(811.1, 813.4)
MH	Summer	5.88	18.2	12.5	1.01	10.2	(9.12, 11.3)	1.70	50.5	(49.4, 51.6)	3.24	1739	(1738, 1740)	3.22	1669	(1668, 1670)
MH	Fall	6.32	18.3	15.5	0.876	7.51	(6.44, 8.58)	1.30	45.9	(44.8, 47.0)	2.93	852.8	(851.7, 853.9)	3.05	1116	(1115, 1117)
PH	Winter	6.89	5.95	20.4	0.740	5.55	(4.55, 6.55)	1.57	36.9	(35.9, 38.0)	2.27	187.6	(186.5, 188.7)	2.21	163.5	(162.4, 164.6)
PH	Spring	7.10	12.2	18.2	0.780	6.03	(5.03, 7.03)	1.62	41.3	(40.2, 42.4)	2.83	672.5	(671.4, 673.7)	2.84	688.8	(687.6, 689.9)
PH	Summer	6.40	26.6	19.8	0.820	6.65	(5.65, 7.65)	1.62	41.8	(40.8, 42.9)	3.17	1489	(1488, 1490)	3.14	1370	(1369, 1371)
PH	Fall	6.71	18.6	21.9	0.770	5.89	(4.83, 6.95)	1.59	38.7	(37.6, 39.7)	2.89	779.7	(778.6, 780.8)	3.09	925.2	(924.1, 926.3)

### Climatic effects

Climatic effects on log_10_ euphotic-layer *chl-a* and log_10_ NPP are expressed as model predictions for low-flow, “dry”, mean-flow, and high-flow, “wet” conditions for OH, MH, and PH salinity zones from 1985 to 2015 (Fig. 10a–f). Simple, linear regressions showed consistent upward trends of log_10_ euphotic-layer *chl-a* for all three salinity zones (Fig. 10a–c). Low-flow conditions did not affect log_10_ euphotic-layer *chl-a* for the OH salinity zone but led to decreased log_10_ euphotic-layer *chl-a* for MH and PH salinity zones. High-flow conditions led to decreased log_10_ euphotic-layer *chl-a* for the OH salinity zone, did not affect log_10_ euphotic-layer *chl-a* for the MH salinity zone, and led to increased log_10_ euphotic-layer *chl-a* for the PH salinity zone. NPP was less sensitive to climatic effects than euphotic-layer *chl-a*, documented as time series of log_10_ NPP for low-flow, mean-flow, and high-flow conditions (Fig. 10d–f). Simple, linear regressions of mean-flow predictions of log_10_ NPP showed upward trends for OH and MH salinity zones, but no trend for the PH salinity zone.

### Euphotic-layer *chl-a*, APPP

Time series of mean, annual euphotic-layer *chl-a* based on observations from 1985 to 2015 showed upward trends for OH and MH salinity zones, but not for the PH salinity zone (Fig. 11a–c). Corresponding APPP estimates from mean-flow model predictions of NPP showed upward trends from 1985 to 2015 for all three salinity zones. APPP ranged from ~200 to 300 g C m^−2^ yr^−1^ for the OH salinity zone, ~250 to 550 g C m^−2^ yr^−1^ for the MH salinity zone, and 280 to 350 g C m^−2^ yr^−1^ for the PH salinity zone. Using Nixon’s trophic classification^3^, APPP corresponded to “eutrophic” conditions for OH and PH salinity zones, and “hypertrophic” conditions for the MH salinity zone. APPP based on mean-flow model predictions of NPP with simulated 40% reductions of euphotic-layer *chl-a* or TN and NO_2_ + NO_3_ loadings showed reductions for all three salinity zones (Fig. 11a–c). These reductions of biomass or nutrient loadings changed APPP to mesotrophic conditions for OH and PH salinity zones, and to eutrophic conditions for the MH salinity zone.

### Historical reconstructions

Archival data on water-quality properties, including P^B^_opt_ from polynomial regressions on SST (Fig. 5a), log_10_ *chl-a*, and other predictor variables (Table 3), supported model predictions of log_10_ NPP in the 1960s and 1970s (Fig. 12a–f). Model predictions from gam4 supported historical reconstructions of log_10_ NPP for low-flow, “dry”, mean-flow, and high-flow, “wet” conditions to capture climatic effects (Table 5). APPP based on mean-flow predictions of NPP for the OH salinity zone reached 555 g C m^−2^ y^−1^ in the 1960s, compared to 288 g C m^−2^ y^−1^ in the 1970s (Fig. 12a,d). APPP for MH and PH salinity zones showed similar patterns, ranging from 392 to 504 g C m^−2^ y^−1^ in the 1960s, and from 132 to 334 g C m^−2^ y^−1^ in the 1970s (Fig. 12b,c,e,f). Mean-flow predictions of log_10_ NPP in the 1960s showed summer maxima for OH and MH salinity zones (Fig. 12a,b), while sparse data for the PH salinity zone limited resolution for that period (Fig. 12c). Observed log_10_ *chl-a* showed summer maxima for OH, MH, and PH salinity zones, and an absence of a spring maximum for the MH salinity zone (Fig. 12b).

## Discussion

### NPP and GPP models

Important advances in NPP and GPP models for estuarine-coastal waters were made possible by increasingly sophisticated approaches and availability of calibration data. Common elements of production models dating to the 1950s include terms for photosynthetic efficiency, phytoplankton biomass, and light availability^41–52^. Light-utilization models specific to estuarine-coastal ecosystems, such as Chesapeake Bay, San Francisco Bay, and mid-Atlantic coastal waters, relied on observations of euphotic-layer *chl-a*, incident irradiance (E_0_), light attenuation coefficient (K_PAR_), NPP, and GPP^11,14,48–50^. In 1997, Behrenfeld and Falkowski developed VGPM, a depth-integrated model applied to ocean-color data from SeaWiFS (Sea-viewing Wide Field of View Sensor) and MODIS (Moderate Resolution Imaging Spectroradiometer) that provide global coverage of NPP^8^. In 2002, we developed the Chesapeake Bay Production Model (CBPM) as the published form of VGPM overestimated NPP and GPP for the bay^7^. Stepwise regressions of log-transformed variables from VGPM led to CBPM that supported estimates of NPP from aircraft and satellite ocean-color data^31,53^.

Here, we departed from VGPM and CBPM to develop production models for the bay using GAM. Selection of predictor variables for NPP and GPP models was informed by earlier studies on a subset of these data from LMER TIES cruises (Table 1). Analysis of variance (ANOVA) showed that season and region explained most of the variability of phytoplankton properties, including NPP, *chl-a*, and floral composition^54^. Long-term (six-year) means of NPP were negatively correlated with the fraction of *chl-a* in diatoms, a property stimulated by high-flow, “wet” conditions. Multiple linear regression and principal component analysis identified SRF as a ‘master variable’ driving inter-annual variability of these properties. An important advantage of transitioning to GAM was the flexibility to include predictor variables such as SRF to adjust for climatic effects on hydrology and distinguish variability from trends.

Model forms developed here were guided by these findings, leading us to include predictor variables for salinity zone, salinity, month, season, year, and flow terms log_10_ SRF and log_10_ SUM (Table 3). These models proved effective to estimate NPP and GPP, exemplified by simple, linear regressions of observed vs. modeled log_10_ NPP from gam2 with R^2^ > 0.96 (Fig. 6a–c), and log_10_ GPP from gam2 with R^2^ > 0.80 (Fig. 6d–f). These fits are comparable to NPP estimates using CBPM with measured values of P^B^_opt_, and GPP estimates using CBPM with estimated values of P^B^_opt_^7^. Predictor variables in models of NPP for OH, MH, and PH salinity zones consistently showed highest F-values and lowest p-values for log_10_ P^B^_opt_ and log_10_ euphotic-layer *chl-a* (Table 3). Several other terms were also significant predictor variables, i.e., TN and NO_2_ + NO_3_ loadings (OH salinity zone), salinity, month (MH salinity zone), and salinity, K_PAR_, SRF, and month (PH salinity zone).

### Climatic effects

Our group has focused on climatic effects on hydrology impacting water quality and phytoplankton in recent studies of Chesapeake Bay^16–19^. Adolf *et al*.^54^ explored this theme previously, reporting predictable consequences of SRF on phytoplankton dynamics. Statistical models based on long-term data extended these findings, documenting climatic effects on *chl-a*, floral composition, and NPP^16–19^. A logical sequence emerged from these studies wherein seasonal to interannual variability of freshwater flow and N loading regulates spatio-temporal distributions of phytoplankton^16–19^, consistent with the conclusion by Malone *et al*.^22^ that P plays a limited, transient role in the OH salinity zone of the bay, while N limits phytoplankton biomass and production on the ecosystem scale.

Despite evidence from shipboard, aircraft, and satellite data linking freshwater flow to phytoplankton dynamics in land-margin ecosystems, previous NPP and GPP models did not contain explicit terms for climatic effects on hydrology^12–14,51,52,54–60^. Analyses described here addressed this shortcoming, based on observations in Chesapeake Bay spanning several decades. Specifically, low-flow, “dry” conditions produce a landward shift of N-limitation toward OH and MH salinity zones, lower *chl-a*, lower NPP, and a lower proportion of diatoms in the phytoplankton flora; high-flow, “wet” conditions extend the area of N sufficiency seaward to MH and PH salinity zones, leading to higher *chl-a*, higher NPP, and a higher proportion of diatoms^16–19,54,60^. Climatic effects on bio-optically active constituents similarly affect light-limitation as higher inputs of dissolved and suspended materials occur for high-flow, “wet” conditions than for low-flow, “dry” conditions^16,18^. This latter observation may contribute to lower sensitivity of NPP than *chl-a* to climatic variability reported here (Fig. 10a–f).

Development of numerical water-quality criteria followed this logic, leading to model predictions that distinguished long-term trends from spatio-temporal variability^18^. Freshwater flow from the Susquehanna River, and frequencies of predominant weather patterns defined “dry” and “wet” conditions^53,60–62^, and statistical models conditioned on specific input terms for flow and salinity supported predictions of mean, monthly *chl-a*, Secchi depth, and NO_2_ + NO_3_^19^. Here, we extended this approach to NPP and GPP models by including terms to adjust for climatic effects on hydrology (Tables 3, 5; Fig. 10a–f). This approach benefited from the flexibility of GAM to incorporate predictor variables traditionally used in production models, i.e., P^B^_opt_, *chl-a* or euphotic-layer *chl-a*, Z_p_, and SST, and to add variables for salinity zone, salinity, season, SRF, and TN and NO_2_ + NO_3_ loadings.

### APPP

Cloern *et al*.^4^ published a synthesis of APPP for estuarine-coastal ecosystems based on a comprehensive survey of the scientific literature. APPP for 131 ecosystems ranged from 105 to 1890 g C m^**−**2^ yr^**−**1^, with a mean of 252 g C m^**−**2^ yr^**−**1^. Ten-fold variability occurred within ecosystems and five-fold from year to year, with only eight time-series covering longer than a decade. One of the best-studied ecosystems in the survey was the Rhode River, a small sub-estuary adjacent to the MH salinity zone of Chesapeake Bay. Long-term measurements of photosynthesis by Gallegos^63^ supported estimates of APPP ranging from 152 to 612 g C m^−1^ y^−1^ in the Rhode River, with a mean of 328 g C m^−1^ y^−1^. APPP maxima occurred in years with dense spring blooms of *Prorocentrum cordatum* (formerly *P. minimum*) a dinoflagellate species that commonly forms “mahogany tides”. Complex interactions of local and remote nutrient inputs affected the relationship of APPP in the Rhode River to SRF. High-flow conditions displaced the turbidity maximum, usually located in the OH salinity zone, south of the Rhode River mouth, causing elevated turbidity in the sub-estuary, washout of phytoplankton, suppression of the spring bloom, and decreased APPP.

Models of NPP calibrated with long-term measurements in Chesapeake Bay from 1982 to 2004 supported multi-year estimates of APPP, based on water-quality properties from 1985 to 2015 as model inputs. These estimates of APPP allowed us to resolve inter-annual variability for a three-decade span, rarely possible for estuarine-coastal ecosystems per Cloern *et al*.^4^. Seasonal to inter-annual variability of NPP and thus APPP can be traced to euphotic-layer *chl-a*, a predictor variable that is highly sensitive to climatic effects on hydrology. We previously related inter-annual variability of APPP to TN and TP loadings, based on the supply of new nutrients from the Susquehanna River during the winter–spring freshet^7^. Stepwise regressions tested time lags between the seasonal pulse of nutrients and maximum NPP in summer, identifying mean, monthly TN and TP loads in February and March as predictors of APPP. Models of NPP developed here used a different approach to capture climatic effects on hydrology, explicitly accounting for variability of freshwater flow and nutrient loadings with predictor variables. The resulting model predictions of NPP supported estimates of APPP, resolving inter-annual variability and long-term trends from 1985 to 2015 (Fig. 11a–c).

### Nixon’s trophic classification, historical context

Mean-flow predictions of NPP were used to estimate APPP from 130 to >600 g C m^−2^ y^−1^ for OH, MH, and PH salinity zones, with increases from 1985 to 2015 matching euphotic-layer *chl-a* (Fig. 11a–c). APPP of this magnitude corresponds to “eutrophic” for OH and PH salinity zones, and “hypertrophic” for the MH salinity zone using Nixon’s^3^ trophic classification (Fig. 1). According to Cloern *et al*.^4^, Chesapeake Bay ranks among estuarine-coastal ecosystems that are heavily impacted by nutrient over-enrichment (their Fig. 4). We evaluated prospects for changing trophic classification based on APPP by simulating 40% reductions of biomass or nutrient loadings in models of NPP. These reductions of euphotic-layer *chl-a* or TN and NO_2_ + NO_3_ loadings led to decreased APPP and changed trophic status from “hypertrophic” to “eutrophic” for the MH salinity zone, and from “eutrophic” to “mesotrophic” for OH and PH salinity zones (Fig. 11a–c).

Simulated 40% reductions of euphotic-layer *chl-a* or TN and NO_2_ + NO_3_ loadings were based on goals established by the 1987 Chesapeake Bay Agreement^64^ to reduce phytoplankton biomass sufficiently to reverse summer anoxia. Several interventions by management began in the 1980s when states bordering the bay banned phosphate in laundry detergents. Subsequent nutrient-management legislation was adopted by Maryland, Virginia, and Pennsylvania in the 1990s, aimed at reducing the over-application of commercial fertilizers and manure on agricultural lands. In 2004, the six states in the watershed, the District of Columbia, and U.S. EPA reached an agreement on comprehensive wastewater treatment permits, leading to numerical annual loading limits for over 470 municipal and industrial wastewater treatment facilities. In December 2010, total manageable daily loads (TMDL) were adopted by U.S. EPA in collaboration with the six states and the District of Columbia. These agreements committed to significant reductions of nutrient and sediment loads by 2025, development of locally based watershed implementation plans, and an accountability system including annual milestones and public reporting of progress.

Together, these actions have led to modest progress toward improved water quality and changes in phytoplankton ecology in the bay^16^, although additional nutrient reductions must be reached to decrease APPP and change trophic classification. Our analyses of long-term trends showed flow-adjusted TN and NO_2_ + NO_3_ loadings doubled from 1945 to 1981, followed by decreases of 19.2% and 5.3% from 1981 to 2012^16^. The slow, upward trajectory of flow-adjusted *chl-a* for the MH salinity zone is consistent with shallow, downward trends of TN and NO_2_ + NO_3_ loadings in recent years^16,18^. We point out that simulated 40% reductions of euphotic-layer *chl-a* or nutrient loadings exceed actual progress since the 1980s, explaining the continuing increases of APPP based on mean-flow model predictions of NPP (Fig. 11a–c).

Decadal contrasts of NPP and APPP in the 1960s and 1970s (Fig. 12a–f) reflected a combination of water-quality management and climatic effects: (1) lower inputs of bio-optically active constituents in the 1960s accompanied a sequence of low-flow, “dry” years compared to the 1970s, reducing light-limitation for OH, MH, and PH salinity zones and enhancing NPP and APPP; (2) removal of orthophosphate (PO_4_^3−^) from detergents enhanced P-limitation in the OH salinity zone, leading to increased N-throughput to MH and PH salinity zones, and reductions of NPP and APPP from the 1960s to the 1970s; (3) model predictions of NPP for low-flow, “dry”, mean-flow, and high-flow, “wet” conditions were based on predictor variables for flow and salinity that adjusted for climatic effects, with mean-flow predictions of NPP and APPP reflecting these adjustments in the 1960s and 1970s.

Model predictions of NPP from historical reconstructions and recent years led to comparable estimates of APPP for the MH salinity zone. Mean-flow model predictions of NPP produced estimates of APPP >500 g C m^−2^ y^−1^ from 2010 to 2015 (Fig. 11b), similar to APPP for the same salinity zone in the 1960s (Fig. 12b). Analogous estimates for OH and PH salinity zones showed a similar pattern, with APPP in the 1960s higher than in recent years. APPP was lower for all three salinity zones in the 1970s than in the 1960s (Fig. 12d–f). These observations and predictions provide historical context for comparison with contemporary conditions, suggesting APPP today is not appreciably different from past rates. We found evidence of lower APPP for MH and PH salinity zones in contemporary estimates, and sensitivity of APPP to reductions of euphotic-layer *chl-a* or TN and NO_2_ + NO_3_ loadings. These findings show promise for future reductions of APPP in response to improvements of water quality that would be required to change trophic classification.

## Summary

Statistical models of NPP and GPP were developed for Chesapeake Bay with adjustments for climatic effects on hydrology, calibrated with 100 s of shipboard measurements from 1982 to 2004. Model predictions of NPP based on water-quality properties in the 1960s and 1970s and 1985 to 2015 as inputs supported computations of APPP. Simulated reductions of euphotic-layer *chl-a* or TN and NO_2_ + NO_3_ loadings led to decreased APPP sufficient to change the trophic classification of the bay. Summarizing:

- Statistical models of NPP and GPP calibrated with long-term data included explicit terms to adjust for climatic effects.
- These models supported predictions of NPP using historical and monitoring data as predictor variables.
- Model predictions of NPP using historical (1960s, 1970s) and monitoring (1985 to 2015) data as predictor variables supported computations of APPP.
- Simulated 40% decreases of euphotic-layer *chl-a* or TN and NO_2_ + NO_3_ loadings reduced APPP and changed trophic classification.
- Improved water quality is attainable with a reversal of nutrient over-enrichment of the bay sufficient to decrease phytoplankton biomass, but progress to date has been modest compared to goals, exemplified by continuing, high APPP in recent years.
